# Effect of *Moringa oleifera* Lam. leaf powder on the pharmacokinetics of nevirapine in HIV-infected adults: a one sequence cross-over study

**DOI:** 10.1186/s12981-017-0140-4

**Published:** 2017-03-14

**Authors:** Tsitsi G. Monera-Penduka, Charles C. Maponga, Alan R. Wolfe, Lubbe Wiesner, Gene D. Morse, Charles F. B. Nhachi

**Affiliations:** 1 Drug and Toxicology Information Services (DaTIS), School of Pharmacy, University of Zimbabwe College of Health Sciences, Harare, Zimbabwe; 2 Center for Integrated Global Biomedical Sciences, University at Buffalo, Buffalo, NY USA; 3 Department of Bioengineering and Therapeutic Sciences, University of California San Francisco, San Francisco, CA USA; 4 Division of Clinical Pharmacology, Department of Medicine, University of Cape Town, Cape Town, South Africa; 5 Department of Clinical Pharmacology, University of Zimbabwe College of Health Sciences, Harare, Zimbabwe

**Keywords:** *Moringa oleifera*, Nevirapine, Pharmacokinetics, HIV

## Abstract

**Background:**

*Moringa oleifera* Lam., an herb commonly consumed by HIV-infected people on antiretroviral therapy, inhibits cytochrome P450 3A4, 1A2 and 2D6 activity in vitro; and may alter the pharmacokinetics (PK) of antiretroviral drugs metabolized via the same pathways. However, in vitro drug interaction activity may not translate to a clinically significant effect. Therefore, the effect of moringa leaf powder on the PK of nevirapine in HIV-infected people was investigated.

**Methods:**

Adult patients at steady-state dosing with nevirapine were admitted for 12-h intensive PK sampling following a 21-day herbal medicine washout. Blood sampling was repeated after 14 days of nevirapine and moringa (1.85 g leaf powder/day) co-administration. Nevirapine plasma concentrations were determined by liquid chromatography-tandem mass spectrometry. To assess the effect of moringa on nevirapine PK, the change in nevirapine area under the plasma concentration–time curve (AUC) was determined. The mean difference in pre- and post-moringa nevirapine, maximum concentration (C_max_) and concentration at 12 h (C_12h_) were also calculated. The PK parameters were compared by assessing the post/pre geometric mean ratios (GMRs) and associated 90% confidence intervals (CIs).

**Results:**

Pharmacokinetics analyses were performed on the results from 11 participants for whom complete data were obtained. The post/pre GMRs and associated 90% CIs for nevirapine were 1.07 (1.00–1.14) for the AUC; 1.06 (0.98–1.16) for C_max_ and 1.03 (0.92–1.16) for C_12h_.

**Conclusion:**

Co-administration of *Moringa oleifera* Lam. leaf powder at the traditional dose did not significantly alter the steady-state PK of nevirapine.

*Trial registration number* NCT01410058 (ClinicalTrials.gov)

## Background

Concomitant use of herbs with drugs may result in herb-drug interactions through various pharmacokinetic and pharmacodynamic mechanisms [1, 2]. The risk of interaction increases with the number of co-administered agents. As a result, HIV patients in developing countries are at high risk of herb-drug interactions because of the widespread self-directed herbal medicine use; and the polypharmacy associated with HIV treatment [3].

A growing number of studies are being conducted to evaluate the pharmacokinetic effects of herbs on drugs when taken together. However, the majority of studies use preclinical in vitro and animal models, which generate data that often do not translate to a clinical effect [4, 5]. In addition, currently available data are skewed towards Chinese and Western herbs. Given the high prevalence of herbal medicine use and the rapid scaling up of treatment for HIV infection in developing countries, rigorous clinical studies are urgently required to assess the effects of commonly used herbal medicines on antiretroviral drugs. This will provide evidence to accurately guide herbal medicine use among HIV-infected people who choose to take herbs and drugs together.

In developing countries, the leaf powder of *Moringa oleifera* Lam. is commonly used as a medicinal herb, rather than food as is the case in Asian populations. It is often taken as a supplement by HIV-infected people to enhance immunity and manage opportunistic infections [6, 7]. In-vitro data suggest that moringa inhibits cytochrome P450 (CYP) 3A4, 1A2 and 2D6 activity which could potentially lead to metabolic interactions with antiretroviral drugs metabolized via the same pathways [5, 8, 9]. The non-nucleoside reverse transcriptase inhibitor nevirapine (200 mg twice daily) is widely used as a component of first line antiretroviral therapy in many developing countries. It is metabolized mainly by CYP3A4 and CYP2B6 and to a lesser extent CYP3A5, CYP2C9, and CYP2D6 [10, 11]. Previous studies have demonstrated that concomitant administration of fluconazole, a potent inhibitor of CYPs, results in markedly increased trough plasma nevirapine concentrations when compared to the administration of nevirapine alone [12]. Concomitant dosing with the herb St. John’s wort, a CYP3A4 and P-glycoprotein inducer, reduced exposure to nevirapine [13]. Such interactions with CYP inhibitors or inducers could potentially increase toxicity [11] or reduce efficacy, respectively. Prior to this study, it was not known whether th*e* in vitro inhibition potential of *Moringa oleifera* Lam. is of clinical significance when co-administered with nevirapine. It was hypothesized that inhibition of CYP3A4 by *M. oleifera* leaf powder may result in a clinically significant increase in nevirapine exposure. This study was conducted to assess the effect of 14 days *Moringa oleifera* leaf powder supplementation on the steady-state pharmacokinetics of nevirapine in HIV-infected patients.

## Methods

### Study participants and setting

HIV-infected male and female adults reporting for routine HIV clinic visits at a referral hospital in Zimbabwe were identified through an interviewer-administered questionnaire. To be enrolled in the study, participants had to be >18 years old, on an nevirapine based regimen for at least 2 weeks and to have supplemented their antiretroviral therapy with herbal medicine of their own accord in the past. Exclusion criteria included anemia (hemoglobin <10 g/dL), impaired renal function (serum creatinine >300 µmol/L) or abnormal hepatic function (serum alanine transaminase >5 times the upper limit of normal) at enrolment, and current treatment with rifampicin, isoniazid or imidazoles. All concomitant medications were also evaluated on a case by case basis and current literature reviewed to exclude any drugs with potential to interact with nevirapine. In addition, female participants were required to have a negative pregnancy test and not to be breastfeeding.

### Preparation and analysis of moringa

Fresh *Moringa oleifera* Lam. leaves positively identified by a botanist from the National Botanical Gardens were harvested from a rural district in Zimbabwe. A voucher specimen was retained and another deposited at the National Herbarium and Botanical Gardens of Zimbabwe (ref. GPS 13504-C). The leaves were then room dried and coarsely ground into powder using a kitchen blender according to the method published by the Moringa Association of Ghana (batch MO001 [14]. The dried leaf powder was assessed for microbial and heavy metal content and a chemical fingerprint of the methanol extract was generated by ultra-performance liquid chromatography (UPLC) coupled to a time of flight mass spectrometer (TOF MS ES+). The fingerprint was archived at the University of Zimbabwe College of Health Sciences.

### Dosage regimen and standardization

In the authors’ experiences and findings from previous surveys, moringa leaf powder is consumed in a wide range of doses [6, 15]. The dose of moringa for the trial was planned at approximately two teaspoons (10 mL) once a day because it was within the reported range and could also be encapsulated into a reasonable number of capsules that would not be a barrier to adherence. This was equivalent to about 1.85 g of the dry leaf powder. To standardize the dose, each 1.85 g dose was weighed out and filled into hard gelatin capsules. This was equivalent to 4 size ‘000’ capsules. Each participant was supplied with enough capsules for the 2-week period.

### Study design and treatments

An open label, two phase, one sequence, cross-over, pharmacokinetic study was conducted over 35 days. The study had a minimum 3-week herbal medication wash out period prior to the first dosing to reduce the possibility of carryover from any type of previously used moringa or other herbal medication, so the participants would have the same baseline. On the first visit (day one), all enrolled participants were instructed not to take any herbal supplements, including moringa. They were also counseled to continue taking their regular antiretroviral drug regimen supplied at the HIV outpatient clinic which included 200 mg nevirapine twice daily. On the second visit (day 22), serial whole blood (5 mL) samples and spot urine sample were collected for determination of baseline plasma nevirapine concentration and urinalysis, respectively. Pre-dose urine and blood samples were collected immediately before an observed nevirapine morning dose. Additional pharmacokinetic blood samples were also collected via cannula at 0.5, 1, 1.5, 2, 3, 4, 6, 8 and 12 h post nevirapine dose. Participants were given further counseling and instructions to continue taking the regular antiretroviral drug regimen supplied at the HIV outpatient clinic together with moringa supplied by the study staff. On the third visit (day 35), a second set of serial whole blood (5 mL) samples and a spot urine sample were collected for determination of post-moringa plasma nevirapine concentration and urinalysis. Pre-dose urine and blood samples were again collected immediately before an observed nevirapine morning dose, and additional pharmacokinetic blood samples collected via cannula at 0.5, 1, 1.5, 2, 3, 4, 6, 8 and 12 h post nevirapine dose. The observed dose of nevirapine was administered in the fed state on both visits. On blood sampling days, a standard diet was provided and participants were required to remain on-site over the course of both 12 h intensive sampling periods.

Documentation of adherence to medication and supplementation restrictions between visits was achieved through the use of a food/herb/medication diary. Exposure to moringa was based on self-reported adherence and assessed through personal interviews, pill counts and evaluation of the food/herb/medication diary.

### Assessment of adverse events

All participants were monitored for adverse events throughout the study. The study medical officer documented any adverse events observed from clinical examinations or self-reported by the participants at each visit.

### Sample preparation

Blood samples were collected into 10-mL tubes containing ethylenediaminetetraacetic acid (EDTA) for determination of nevirapine concentration. All blood specimens were centrifuged at 2800×*g* for 5 min at 4 °C. The plasma was harvested and stored at −80 °C until time of analysis.

### Determination of nevirapine

Nevirapine concentrations were determined by LC–MS/MS in the laboratory of the Division of Clinical Pharmacology, University of Cape Town. The assay method has been reviewed by the AIDS Clinical Trials Group Quality Assurance and Quality Control program (ACTG CPQA). It was validated according to the United States Food and Drug Administration and European Medicines Agency guidelines using externally prepared proficiency testing samples supplied by the ACTG CPQA.

Plasma samples were extracted from 100 µL human plasma by protein precipitation using acetonitrile (Honeywell, Burdick & Jackson^®^). The extraction procedure was followed by liquid chromatography with MS/MS detection. Chromatographic separation was achieved on a Luna 5 μm PFP (2), 100 A, 50 mm × 2 mm analytical column column (Phenomenex, Torrance, CA, U.S.). An AB Sciex API 4000 mass spectrometer (SCIEX, Framingham, MA, U.S.) was operated at unit resolution in the multiple reaction monitoring (MRM) mode, monitoring the *m/z* 266.9 (MH^+^) → 198.2 transition for nevirapine, and the *m/z* 270.1 → 229.1 transition for the deuterated nevirapine internal standard. The calibration curve was fitted by quadratic regression (weighted by 1/concentration^2^) over the range 0.0195–20.0 µg/mL. The combined accuracy and precision statistics of the low, medium and high quality control samples during sample analysis were between 89.8 and 93.4%, and 4.2 and 9.1%, respectively.

### Statistical and pharmacokinetic analysis

In designing the study, a difference in pre- and post-moringa nevirapine area under the plasma concentration–time curve from 0 to 12 h (AUC_0–12h_) of at least 20% was considered to be clinically relevant for the purpose of establishing sample size. An intra-individual coefficient of variation of 25% was assumed for nevirapine AUC based on previously published data [16]. With α = 0.05, it was determined that a total of 12 participants would provide 80% power in the case of an equivalence range 0.8–1.25 [17]. After making adjustments for loss to follow-up (10%) and the cross-over design, a sample size of 13 was targeted.

Statistical analyses were performed using StataMP^®^ version 13 (StataCorp, College Station, TX). Demographic characteristics were summarized as means and standard deviations or frequencies. Nevirapine pharmacokinetic parameters [i.e. AUC_0–12h_, peak concentration (C_max,ss_) and concentration at 12 h post dose (C_12h_)] were calculated for each individual using a non-compartmental approach by means of the Phoenix WinNonlin software application (Version 6.0; Certara, Princeton, NJ). AUC was calculated using the linear trapezoidal method. The individual differences in the pharmacokinetic parameters were calculated on log transformed data. The confidence interval approach was used to assess the effect of moringa on the pharmacokinetics of nevirapine. To evaluate if pharmacokinetic changes could have clinical relevance, it is recommended to treat pharmacokinetic interactions as equivalence problems [18]. Absence of a moringa effect was assumed if the 90% confidence interval (CI) of the geometric mean ratio (GMR) of AUC_0–12h_, C_max,ss_ and C_12h_ with and without moringa were contained within the limits 80–125%. The significance of the GMR results were assessed by paired Student *t* test.

Post-moringa/pre-moringa geometric mean ratios (GMRs) and their confidence intervals were calculated as follows: GM[x] = 10^mean[log[x]]; GMR[y/x] = GM[y]/GM[x]. The 90% confidence interval (CI) of GMR[t2/t1] was calculated from the difference in the logs (DL) of the t2 and t1 values: DL[t2,t1] = log[t2]–log[t1], where t2 and t1 are corresponding values for the same subject post-moringa and pre-moringa, for 11 subjects. If m = mean[DL[t2,t1]], sd = standard deviation [DL[t2,t1]], and (a,b) = CI[m,sd,n], then the CI of GMR[t2/t1] = (10^a,10^b) and GMR[t2/t1] = 10^m. The confidence intervals of the DL values were also verified with that obtained by the statistical package.

## Results

### Participant enrolment

Thirty-three HIV-infected adult patients were screened. Thirteen participants met the eligibility criteria and were enrolled after giving oral and written informed consent. All participants completed the study. Based on pill counts and a review of entries in the food/herb/medication diaries, all participants were fully compliant with medication and supplementation restrictions. The baseline demographic characteristics are summarized in Table 1.

**Table 1:** Baseline characteristics of the study population, n = 13

Characteristic	Value
Age (years), mean ± SD	44 ± 8
Sex, female, n (%)	8 (73)
Weight (kg), mean ± SD	63.5 ± 7.5
Drug frequency n (%)	
Nevirapine	13 (100)
Tenofovir/lamivudine	10 (77)
Stavudine/lamivudine	2 (15)
Zidovudine/lamivudine	1 (8)
Cotrimoxazole	9 (69)
Other drugs^a^	3 (23)

^a^Other drugs included pyridoxine, hydrochlorothiazide, indomethacin, diclofenac and enalapril

### Adverse events

The combination of moringa with nevirapine was well tolerated. Participants were in good health upon physical examination at the post moringa visit. There were no serious adverse events and all participants completed the study. A total of two adverse events were reported by the participants; one complained of transient mild pain at the site of cannula insertion and the other, a headache. During the observation period, only grade 1 Common Terminology Criteria for Adverse Events (CTCAE) toxicities were observed in the clinical assessments. The incidence rate was 5% (n = 202 readings), and there were no significant differences in the number observed pre-moringa compared to post-moringa. The medical officer and consultant physician monitoring adverse events in the study decided these were not related to the moringa.

### Pharmacokinetics

Pharmacokinetic analyses were performed on the results from 11 participants who had complete data. Of the two participants that were excluded, one had difficulties with venous access at points during visit 2. The other took the day 35 nevirapine dose at home instead of at the clinic such that only samples for the latter time points could be collected.

**Fig. 1:**
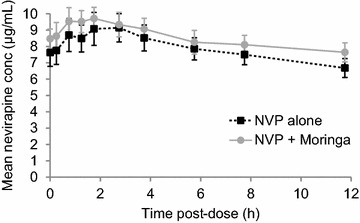
Mean nevirapine plasma concentration–time profile with and without moringa. The *error bars* represent the standard error of the mean

**Fig. 2:**
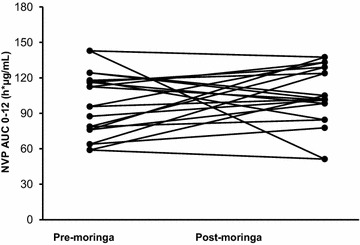
Individual AUC _0–12_ of nevirapine with and without moringa. All patients maintained therapeutic plasma concentrations (>3.0 µg/mL) 12 h post-dose [19]

**Fig. 3:**
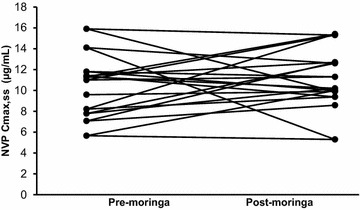
Individual Cmax,ss of nevirapine with and without moringa. All patients maintained therapeutic plasma concentrations (>3.0 µg/mL) 12 h post-dose [19]

**Fig. 4:**
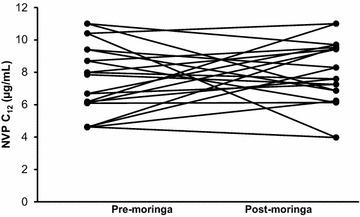
Individual C_12_ of nevirapine with and without moringa. All patients maintained therapeutic plasma concentrations (>3.0 µg/mL) 12 h post-dose [19]

Figure 1 shows the mean plasma concentration–time profiles for nevirapine with and without *Moringa oleifera* leaf powder supplementation (Figs. 2, 3, 4).

The plasma pharmacokinetics parameters and comparisons for nevirapine are presented in Table 2.

**Table 2:** Comparison of nevirapine pharmacokinetic parameters pre- and post- moringa

Parameter	GM (CV %)^a^		GMR (90% CI)^a^	
	Pre-moringa	Post-moringa	Post/Pre	P
AUC_0–12h_ (h*µg/mL)	94.17 (25.24)	100.44 (27.65)	1.07 (1.00–1.14)	0.131
C_12h_ (µg/mL)	7.30 (25.85)	7.55 (28.56)	1.03 (0.92–1.16)	0.702
C_max,ss_ (µg/mL)	9.92 (26.93)	10.57 (29.62)	1.06 (0.98–1.16)	0.261

^a^Post-moringa/pre-moringa geometric mean ratios (GMRs) and their confidence intervals were calculated as follows: GM[x] = 10^{m} ean[log[x]];GMR[y/x] = GM[y]/GM[x]

*GMR* geometric mean ratio, *CI* confidence interval, *AUC*_*0*–*12h*_ area under the time-concentration curve from 0 to 12 h after dosing, *C*_*max,ss*_ maximum concentration, *C*_*12h*_ concentration at the end of the dosing interval

## Discussion

Based on previous in vitro studies that have noted significant inhibitory effects of moringa on the nevirapine-metabolizing CYP3A4 and CYP2D6 isoforms, the effect of *Moringa oleifera* leaf supplementation on nevirapine pharmacokinetics was investigated. While the nevirapine pharmacokinetic profiles from HIV-infected adults at the dosage of moringa used show an inhibitory trend, consistent with that observed in vitro [5, 8, 9], the change in steady-state nevirapine pharmacokinetic parameters is neither clinically nor statistically significant. The 90% confidence intervals for the nevirapine geometric mean ratios for AUC_0–12h_, C_max,ss_ and C_12h_ following concomitant administration of moringa over 14 days were within the 80–125% limit, indicating lack of a clinically significant interaction.

The findings are supported by observations from previous studies that demonstrated that the inhibitory effects of moringa on CYP3A4 and CYP2D6 are significantly less as compared to their positive control inhibitors ketoconazole and quinidine [20]. Data from other studies show that nevirapine induces its metabolism by both CYP3A4 and CYP2B6, which may compensate to some degree for CYP3A4 inhibition [21] Any inhibition of CYP3A4 may also have been compensated for by the alternative metabolic pathways (CYP2B6, CYP3A5, CYP2C9, and CYP2D6) reducing the resulting pharmacokinetic changes [22, 23]. This may not be the case for other drugs such as saquinavir, ritonavir, indinavir, darunavir and atazanavir whose main or sole route of elimination is via CYP3A4.

In general, moringa has a good safety profile consistent with its long history of use as food and medicine [24]. The combination of moringa and nevirapine in this study was also well tolerated since moringa did not alter the safety profile of nevirapine when co-administered. A minimum concentration of 3.0 µg/mL is recommended as a therapeutic cut-off for nevirapine trough concentrations [19]. All patients maintained nevirapine 12-h concentrations above 3.0 µg/mL with moringa supplementation. Because the study did not assess clinical outcomes, this observation cannot be discussed in terms viral load.

The toxicity of nevirapine as a function of concentration is not well established. Some studies could not find evidence of a concentration—toxicity relationship while others report an association between hepatotoxicity and higher nevirapine concentrations [25–28]. In this study, no nevirapine related adverse effects were reported despite the C_12h_ and C_max,ss_ values observed being mostly higher than the 6 µg/mL value previously associated with increased toxicity. The findings are consistent with Thai and Ugandan data that did not detect increased toxicity, despite increases in nevirapine C_trough_.

One limitation of the study is that for operational economy, a single sequence of moringa administration was used; hence, potential period effects cannot be ruled out.

## Conclusions

We conclude that co-administration of moringa leaf powder at traditionally used doses has no clinically significant effect on the steady-state pharmacokinetics of nevirapine.
